# Protein Carbonyl as a Biomarker of Oxidative Stress in Severe Leptospirosis, and Its Usefulness in Differentiating Leptospirosis from Dengue Infections

**DOI:** 10.1371/journal.pone.0156085

**Published:** 2016-06-09

**Authors:** Narmada Fernando, Shalini Wickremesinghe, Roshan Niloofa, Chaturaka Rodrigo, Lilani Karunanayake, H. Janaka de Silva, A. R. Wickremesinghe, Sunil Premawansa, Senaka Rajapakse, Shiroma M. Handunnetti

**Affiliations:** 1 Institute of Biochemistry, Molecular Biology and Biotechnology, University of Colombo, Colombo 03, Sri Lanka; 2 Tropical Medicine Research Unit, Faculty of Medicine, University of Colombo, Colombo 08, Sri Lanka; 3 National Reference Laboratory for Leptospirosis, Medical Research Institute, Colombo 08, Sri Lanka; 4 Faculty of Medicine, University of Kelaniya, Kelaniya, Sri Lanka; 5 Faculty of Science, University of Colombo, Colombo 03, Sri Lanka; Kermanshah University of Medical Sciences, ISLAMIC REPUBLIC OF IRAN

## Abstract

Pathogenesis of disease severity in leptospirosis is not clearly understood whether it is due to direct damage by pathogen or by adverse immune responses. Knowledge on biomarkers of oxidative stress which could be used in identifying patients with severe illness has shown to be of great value in disease management. Thus, the main aim of this study was to assess the damage to serum proteins and lipids, and their significance as biomarkers of oxidative stress in severe leptospirosis. In regions endemic for both leptospirosis and dengue, leptospirosis cases are often misdiagnosed as dengue during dengue epidemics. Therefore, the second aim was to assess the potential of the oxidative stress markers in differentiating severe leptospirosis from critical phase dengue. We measured serum antioxidants (uric acid and bilirubin), total antioxidant capacity (AOC), protein carbonyl (PC) and lipid hydroperoxide (LP) in patients with severe leptospirosis (n = 60), mild leptospirosis (n = 50), dengue during the critical phase (n = 30) and in healthy subjects (n = 30). All patient groups had similar total antioxidant capacity levels. However, the presence of significantly high uric acid and total bilirubin levels may reflect the degree of renal and hepatic involvement seen in severe leptospirosis patients (p<0.02). Serum PC and LP levels were significantly higher in leptospirosis patients compared to critical phase dengue infections (p<0.005). Moreover, high serum PC levels appear to differentiate SL from DC [area under the curve (AUC) = 0.96; p<0.001]. Serum PC may be a reliable biomarker of oxidative damage to serum proteins to identify severe leptospirosis patients (AUC = 0.99) and also to differentiate severe leptospirosis from mild cases (AUC = 0.78; p<0.005) indicating its contribution to pathogenesis. Use of serum PC as an indicator of leptospirosis severity and as an oxidative stress biomarker in differentiating leptospirosis from dengue would provide the opportunity to save lives via prompt patient management.

## Introduction

Leptospirosis is one of the most common zoonotic infections worldwide which causes high annual deaths [1–3]. The major burden of the disease is carried by developing countries. Leptospirosis contributes to a significant proportion of hospitalized cases of febrile illnesses in endemic areas, with 5–10% of mortality [2, 4]. In areas where other acute febrile illnesses, such as dengue and malaria are common, the clinical diagnosis of leptospirosisis is often a challenge, because clinical manifestations in the early stages of the disease are relatively non-specific [5]. The majority of infections are asymptomatic; a few patients go on to develop severe forms of leptospirosis characterised by acute kidney injury, pulmonary haemorrhage, bleeding manifestations, myocarditis, and sometimes multi-organ dysfunction [6]. The pathogenesis of severe forms of leptospirosis is poorly understood. Both pathogen and host factors are thought to be responsible for the development of severe disease. However, it is not clearly known whether severe illness is a result of direct tissue damage caused by the pathogen, or whether it is a consequence of a deranged immune response to infection [3].

During the respiratory burst, reactive oxygen species (ROS) (superoxide anions, hydroxyl radical, singlet oxygen and hydrogen peroxide) are produced in large amounts [7]. This enhanced expression of inducible nitric oxide synthase and pro-inflammatory cytokines results in increased production of reactive nitrogen species (RNS) (i.e., nitric oxide, nitrites and nitrates), and pro-inflammatory cytokines. ROS and RNS are primarily involved in killing pathogens and therefore are beneficial to the host. However, production of large amounts may result in damage to host tissues as well [8].

ROS and RNS cause oxidation of proteins, commonly the amino acids; lysine, arginine and proline. Oxidative cleavage of the peptide backbone via the α-amidation pathway, oxidative cleavage of glutamyl residues, formation of protein-protein cross-linked derivatives, and cell membrane damage by lipid oxidation products give rise to reactive aldehydes and ketones known as protein carbonyls [9–11]. Protein carbonyl (PC) content in blood and tissues is a reliable indicator of protein oxidation. It is particularly useful in this respect because of its long lasting stability under suitable storage conditions (i.e., -80°C) [12]. PC is the most commonly used, and also the most general indicator of oxidative protein damage [13, 14].

ROS and RNS initiate lipid peroxidation by abstracting a hydrogen atom from polyunsaturated fatty acids [15]. Although polyunsaturated fatty acids are the main targets [16], unsaturated phospholipids, glycolipids and cholesterol are also highly susceptible to oxidation by pro-oxidants [7]. Oxidative damage to lipids can occur either by degradation or by decomposition of lipids. Lipid hydroperoxide (LP) levels are an overall index of total lipid peroxidation [17].

Anti-oxidants are reactive agents which detoxify both intracellular and extracellular ROS and RNS, providing protection against them. Glutathione, albumin, uric acid and bilirubin are common anti-oxidants, important in maintaining the balance between pro-oxidants and anti-oxidants in healthy states. During infective/inflammatory processes, or as a part of ageing, there is a shift in the delicate balance between anti-oxidants and pro-oxidants. Increased amounts of ROS and RNS in the blood cause anti-oxidants to be depleted, resulting in diminished anti-oxidant capacity [18]. As the severity of illness increases, anti-oxidants are depleted to a greater extent [19]. Although measurement of free radicals is complicated *invivo*, anti-oxidant capacity (AOC) can be measured in serum, and is an indirect indicator of both ROS and RNS production and the potential for overall protection against oxidative damage [20].

Oxidative stress is known to be responsible for tissue damage in other diseases. For example, in severe sepsis, it has been demonstrated that increased production of ROS and RNS together with depletion of anti-oxidants results in increased oxidative stress and this is associated with tissue injury and disease severity[18].Similar findings have been recorded in human leptospirosis [18, 21–23]. It is thought that the pathogenesis of leptospirosis is significantly different from bacterial sepsis.

In areas where the incidences of both dengue and leptospirosis are high, clinical differentiation of the two conditions is often a challenge. It is well known that, leptospirosis cases are often misdiagnosed as dengue in areas which are endemic for both diseases [24, 25]. The difficulty of clinical differentiation of leptospirosis from dengue emphasizes the requirement of practically usable effective biomarkers in disease differentiation.

The broad objective of our study was to determine the role of oxidative stress in the pathogenesis of severe forms of leptospirosis. Our first aim was to determine the levels of protein carbonylation, lipid hydroperoxidation in serum of patients with leptospirosis and to measure the levels of individual anti-oxidants and the cumulative anti-oxidant capacities. Our second aim was to assess the use of these markers to identify patients with severe forms of leptospirosis and to differentiate severe leptospirosis patients from critical phase dengue infections. Finally, analyses were carried out to study the association between these markers and specific disease complications and laboratory parameters in patients with leptospirosis.

## Materials and Methods

### Recruitment of patients

Patients admitted to the National Hospital of Sri Lanka (NHSL) and Base Hospital, Homagama, Sri Lanka, who were clinically suspected of having leptospirosis were recruited during the period from July 2013 to July 2014. The clinical criteria to define probable cases of leptospirosis were adopted from the World Health Organization (WHO) surveillance criteria [26]. Acute febrile illness with headache, myalgia and prostration plus (conjunctival suffusion or anuria or oliguria/haematuria or jaundice or cough, haemoptysis and breathlessness or haemorrhages or meningeal irritation or cardiac arrhythmia or failure or skin rash) or with history of exposure to *Leptospira* were considered as the clinical criteria for inclusion. At the point of recruitment, patients clinically suspected for having leptospirosis, but with definite alternative diagnosis of dengue, influenza, meningitis, hepatitis or any other confirmed infections were excluded. Clinical investigations, haematological and biochemical data of patients were recorded daily until the discharge of the patient or the death. Blood samples were collected on the day of recruitment and a convalescent sample was also collected on day 21 of illness.

### Controls

Participants from the community, with confirmed negative results for diagnosis of leptospirosis (based on the microscopic agglutination test [MAT]) were selected as healthy controls. To avoid the dietary effect on the base levels of parameters tested in healthy controls, participants were requested to fast for 12 hours prior to blood sampling. Patients with dengue were also recruited during the critical phase of illness (definition based on WHO guidelines for dengue management [27]), as a comparative group.

Serum samples collected from all patients and control subjects were analyzed within a month from the day of collection.

### Laboratory confirmation and clinical categorization in leptospirosis

MAT was performed at the Medical Research Institute, Colombo (the national reference laboratory for this test) for laboratory confirmation of the leptospirosis. MAT positivity was considered as one of the following: MAT titre ≥ 1:400 in single or paired serum samples or four-fold increase in MAT titer between acute and convalescent serum samples, or sero-conversion to ≥ 200 in MAT titre [28]. Laboratory confirmed leptospirosis patients with positive results for dengue non-structural protein 1 (NS1), anti-dengue IgM and anti-hanta IgM antibodies were excluded. Patients, clinically diagnosed and laboratory confirmed to have leptospirosis were further categorized as severe or mild using specific clinical and biochemical parameters, i.e., jaundice (bilirubin>51.3 μmol/L), renal insufficiency (oliguria, urine output <400 ml per day), creatinine>133 μmol/L), urea >25.5 mmol/L and indicators of poor outcome (stay in intensive care unit, death, dialysis, prolonged hospital stay >10 days and multiple organs involved) [29, 30].

### Laboratory confirmation of dengue

Serum samples were tested for dengue NS1 antigen during the febrile phase of the fever using the commercially available SD BIOLINE Dengue NS1 antigen kit (Standard Diagnostics Inc., South Korea) according to the manufacturer’s instructions [31]. Appearance of a band in the test window was considered as a positive result. A second sample was collected during the critical phase of laboratory confirmed dengue patients for further analysis.

### Determination of serum protein carbonyl levels

Serum protein levels were measured and the concentration was brought to 0.5–2.0 mg protein in the test sample by diluting with high purity water (TKA MicroMed, TKA Wasseraufbereitungssysteme GmbH, Germany). Protein carbonyles (damaged proteins) in the serum were measured using a commercially available protein carbonyl content kit (BioVision Inc., USA) according to the manufacturer’s instructions.

### Determination of serum lipid hydroperoxide levels

LP levels were measured using the ferrous oxidation–xylenol orange (FOX2) assay. In acidic medium, lipid hydroperoxides oxidize iron(II) to iron(III). The resulting iron(III) reacts with the dye xylenol orange [o-cresolsulfonphthalcin-3,3-bis(methyliminodiacetic acid sodium salt)] in equal molar concentration and produces a blue-purple colour precipitate [32].

Serum samples were divided into 4 aliquots of 45 μL, each in microcentrifuge tubes and 2 were treated with 10 mM tryphenylphosphine (TPP) and the other two were treated with HPLC grade methanol. Ammonium ferrous sulphate and xylenol orange were immediately dissolved in 250 mM sulfuric acid to prepare FOX2 reagent 450 μL of FOX2 reagent was added to each tube after 30 min, again kept at room temperature for 30 min in dark. Tubes were then centrifuged at 15,000 *g* for 10 min at 20°C and the absorbance of the supernatant was measured at 560 nm using spectrophotometer (Synergy HT multimode microplate reader, Biotek, USA). Sera treated with TPP served as the controls of each sample by oxidizing hydroperoxides. TPP specifically reduces hydroperoxides and the absorbance of the TPP treated sera were due to the direct reaction between serum ferric ions and xylenol orange. Therefore the absorbance due to the actual level of hydroperoxide in a sample was obtained using the following equation, (Absorbance_corrected_ = Absorbance_methanol_- Absorbance _TPP_). LP levels of test samples were extrapolated using the Absorbance_corrected_ from the standard curve of H_2_O_2_concentrations.

### Determination of serum cumulative anti-oxidant capacity

Serum AOC levels were tested using 2,2'-azinobis-(3-ethylbenzothiazoline-6-sulfonic acid) (ABTS) decolourization method, expressed as serum 6-Hydroxy-2,5,7,8-tetramethyl-chroman-2-carboxylic acid (Trolox) equivalent antioxidant capacity (TEAC) [33]. Radical cations were generated by the oxidation of ABTS with potassium persulfate (K_2_S_2_O_8_). ABTS working solution was prepared by diluting the freshly prepared ABTS stock solution in 40 fold with 5 mM phosphate buffered saline (PBS). Distilled water and K_2_S_2_O_8_ in equal volumes (10 μL in each) were mixed with 800 μL of 5 mM PBS to prepare reagent blank. Test sera were mixed with ABTS working solution in 1: 9 ratio and kept for 1 min in dark to complete the scavenging process. Samples were analyzed in duplicates and absorbance were measured at 734 nm against the reagent blank using spectrophotometer (Synergy HT multimode microplate reader, Biotek, USA) [34, 35]. A series of Trolox two fold dilutions (400–12.5 μM) were mixed at the same ratio with ABTS working solution and the standard curve was plotted using absorbance values. TEAC was calculated using the Trolox standard curve.

#### Measure of serum protein levels

Total serum protein levels of patients and healthy controls were determined by measuring the absorbance of diluted serum (100 fold with 0.13 M PBS) at 260 and 280 nm using spectrophotometer [36] (Synergy HT multimode microplate reader, Biotek, USA). TEAC was corrected for serum protein level as serum protein concentrations vary from individual to individual.

### Determination of serum uric acid and total bilirubin levels

Serum uric acid levels (UA) of patients with leptospirosis and dengue and control samples were determined using a commercially available uric acid kit (BioMaxima S.A., Poland). Serum total bilirubin levels (TB) were also measured using a commercially available kit (Randox Laboratories Limited, UK) according to the manufacturer’s instructions.

### Ethical statement

Ethics approval was obtained from the Ethics Review Committee of Faculty of Medicine, University of Colombo (EC-12-056). Informed written consent was obtained from all the participants prior to recruitment to the study.

### Statistical analysis

Statistical analysis was performed using Statistical Package for the Social Sciences/ Statistical Product and Service Solutions (SPSS) 17.0 version. Descriptive statistics were summarized as percentages and mean ± SD. Multivariate regression, one-way ANOVA with Bonferonni post hoc correction, linear regression, Pearson correlation and receiver operating characteristic (ROC) were done where needed. Statistical significance was defined as p < 0.05 at confidence interval of 95%.

## Results

### Study population and patient categorization

Out of 140 patients with acute febrile illness;110 were laboratory confirmed for having leptospirosis (CL) based on MAT while the other 30 were confirmed as dengue cases who developed critical phase of the illness (DC). Of the 110 CL patients, 60 patients were identified as having severe leptospirosis (SL) and the rest (n = 50) as mild leptospirosis (ML) based on clinical categorization. Thirteen SL and 17 ML patients were considered as laboratory confirmed cases (CL) only after performing MAT on the convalescent samples, as the MAT titre in acute sample was less than 400.

In addition, there were 30 age- and gender- matched healthy controls (HC) fasted for 12 hours prior to sampling. Primary demographic data of these groups are shown in Table 1.

**Table 1:** Comparison of demographic and temporal parameters among study groups.

Demographic and temporal parameter	Study group			
	Severe Leptospirosis	Mild Leptospirosis	Critical phase Dengue	Healthy controls
Sample size	60	50	30	30
Age ^a^	40.6 ± 14.0	37.2±14.9	36.2±8.2	38.1±12.2
Gender (Male: Female)	53: 7	43: 7	26: 4	26: 4
Day of illness on sampling day ^a^	8.5±2.9	6.6±2.7	5.3±1.6	-
Days under medication till sampling day ^a^	3.1±2.6	2.1±1.6	3.3±1.4	-

^a^ -Mean ± Standard deviation

### Evidence of increased oxidative stress in leptospirosis

The levels of carbonylated proteins, hydroperoxidated lipids, individual anti-oxidants and cumulative anti-oxidant capacities of laboratory confirmed leptospirosis patients (both SL and ML; n = 110), severe leptospirosis patients (SL; n = 60), mild leptospirosis patients (ML; n = 50), patients in critical phase of dengue infections (DC; n = 30) and healthy controls (HC; n = 30) are presented in S1 Table.

**Fig 1:**
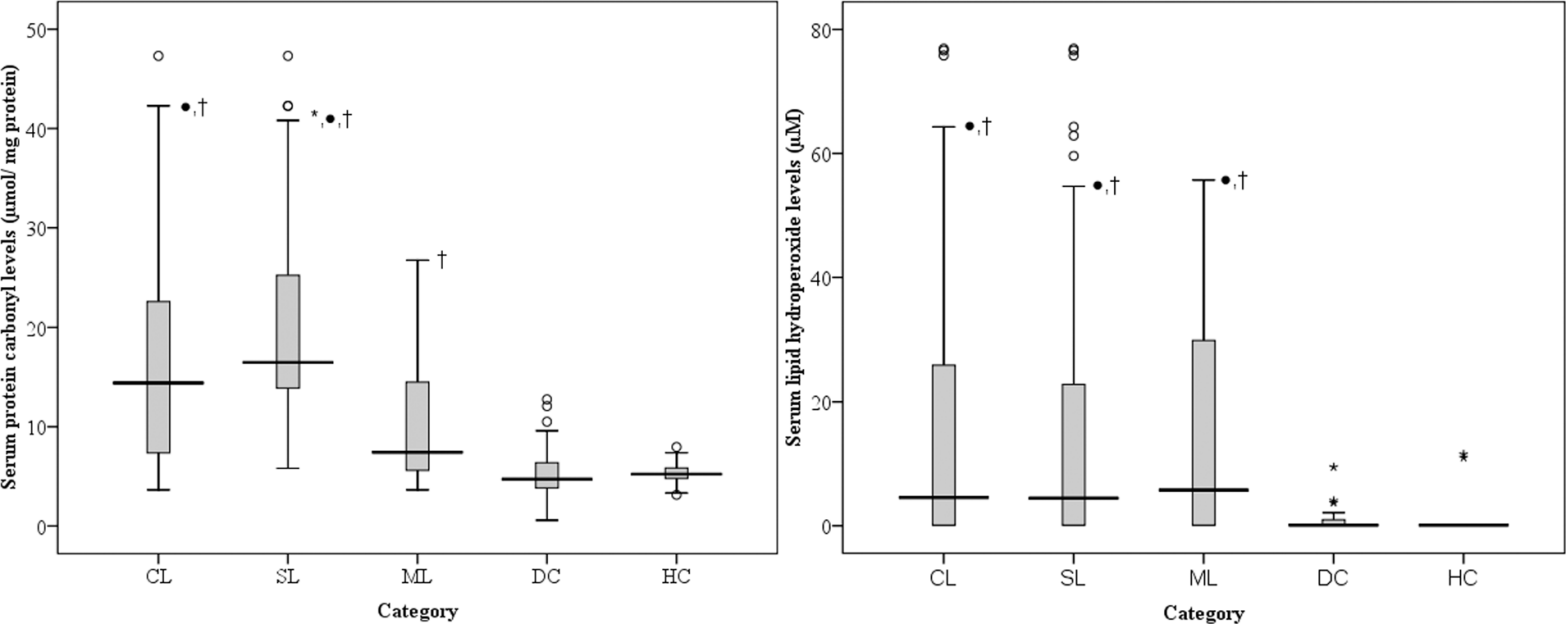
Levels of carbonylated serum proteins and hydroperoxidated lipids. (A) Serum protein carbonyl levels (μmol/ mg protein) and (B) serum lipid hydroperoxide levels (μM) in CL (n = 110; collectively both SL and ML), SL (n = 60), ML (n = 50), DC and HC (n = 30/ group). *—p<0.05 between SL and ML, ●—p<0.05 between any of leptospirosis category (CL, SL and ML) with DC respectively, †—p<0.05 between any of patient category (CL, SL, ML and DC) with HC respectively. The intra-assay coefficient of variability (% CV) was 1.9% and inter-assay % CV was 0.6% for the FOX2 assay, indicating high reproducibility of the test in the laboratory conditions.

CL patients had significantly higher serum PC levels and LP levels compared to HC (p<0.005), indicating the presence of high levels of oxidised serum proteins and lipids (Fig 1).

**Fig 2:**
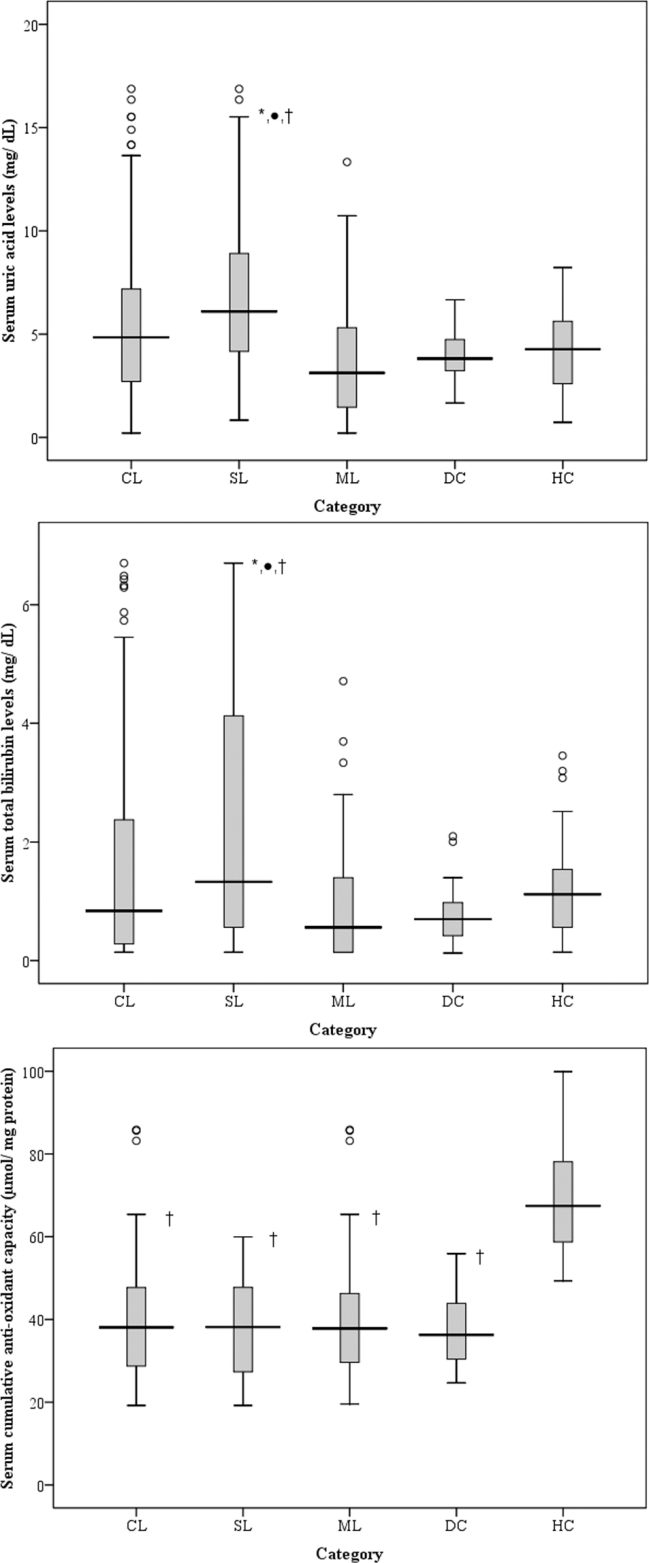
Serum uric acid, total bilirubin and cumulative anti-oxidant capacities. (A) Serum uric acid levels (mg/ dL), (B) serum total bilirubin levels (mg/ dL) and (C) Serum anti-oxidant capacity (μmol/ mg protein) among CL (n = 110; collectively both SL and ML), SL (n = 60), ML (n = 50), DC and HC (n = 30/ group). *—p<0.05 between SL and ML, ●—p<0.05 between any of leptospirosis category (CL, SL and ML) with DC respectively, †—p<0.05 between any of patient category (CL, SL, ML and DC) with HC respectively. The ABTS decolourization method had good intra-assay % CV (7.9%) and inter-assay % CV (6.9%).

Cumulative anti-oxidant capacity was significantly low in CL over HC (p<0.001). However, no significant differences were observed in UA or TB levels of CL and HC groups (Fig 2).

### Identification of patients with leptospirosis from dengue infections

**Fig 3:**
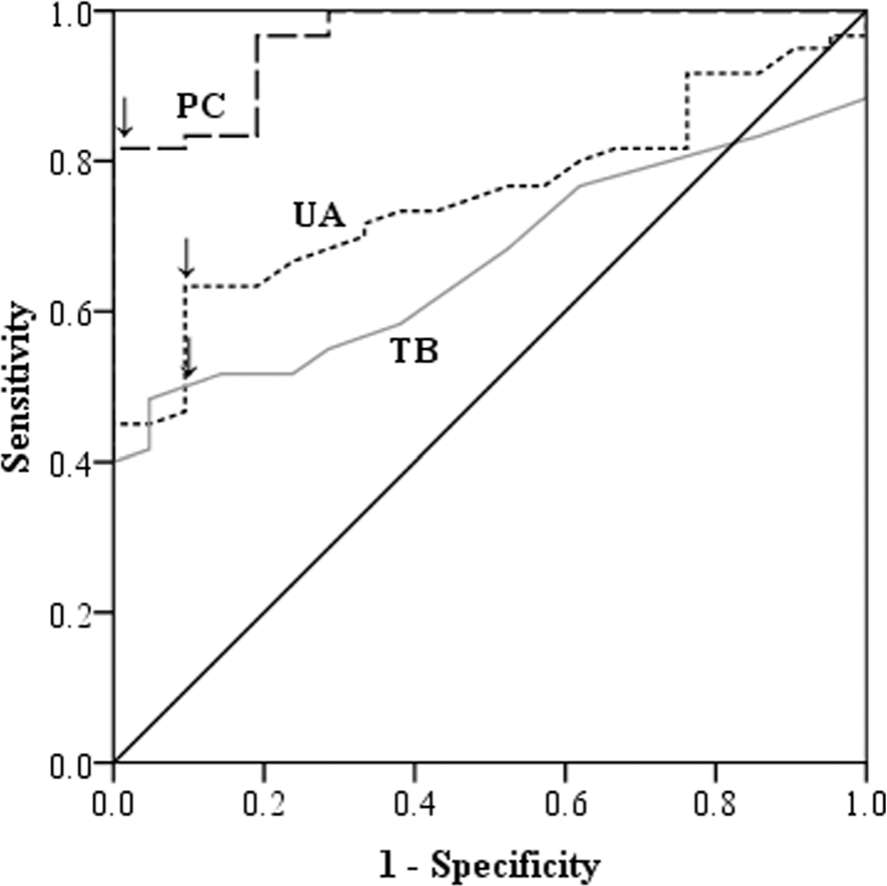
Receiver operating characteristic curves of protein carbonyl levels between severe leptospirosis and critical phase dengue patients. Arrow head shows the cut-off of 13 μmol/mg protein.

CL patients had significantly higher serum PC levels compared to DC (p<0.005). ROC analysis between SL and DC shows that the test had 100% specificity and 82% sensitivity for the cut-off of 13 μmol/ mg protein of PC level (AUC = 0.96; p<0.001). Also, UA and TB had high specificities (90.5% in both) and moderate sensitivities (63% and 50% respectively) in identifying a SL case over DC (Fig 3).

### Differentiation of patients with severe leptospirosis from mild leptospirosis using oxidative stress markers

**Fig 4:**
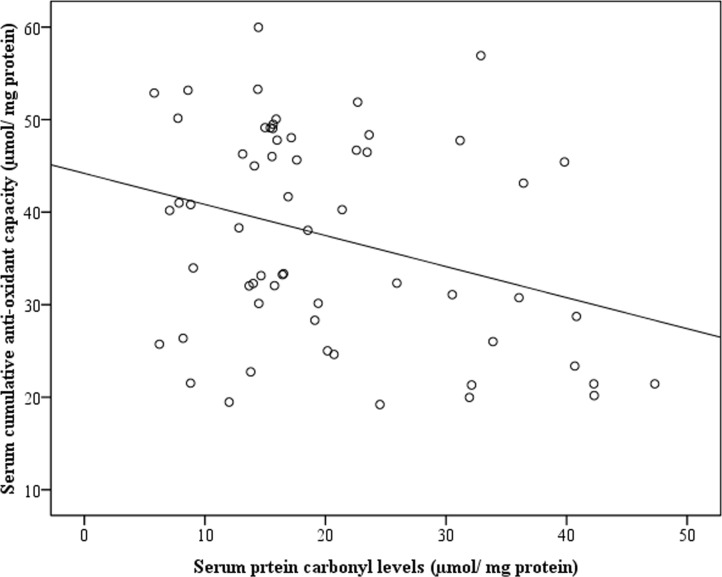
Cumulative serum anti-oxidant capacities in patients with severe leptospirosis with respect to serum protein carbonyl levels(r = 0.312; p = 0.015).

Among CL patients, SL patients had significantly higher PC levels compared to ML patients (p<0.001; Fig 1A). Unlike PC, there was no difference in serum LP levels between SL and ML patients(Fig 1B).Serum UA levels (Fig 2A) and TB levels (Fig 2B) were significantly higher in SL patients compared to ML (p<0.005). Serum PC levels negatively correlated with serum AOC levels in SL patients. Hence, an increase of PC by 1 μm/ mg protein correlated with depletion of AOC by 0.29 μm/ mg protein (r = 0.312; p = 0.015) (Fig 4).

**Fig 5:**
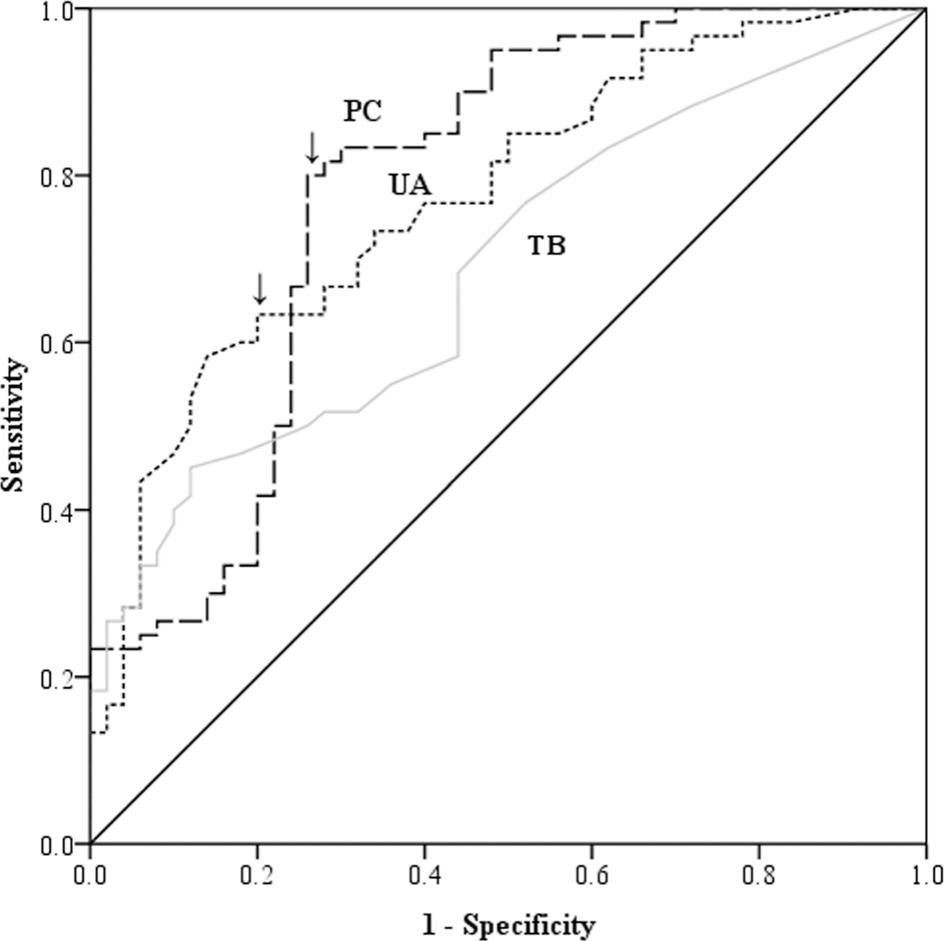
Receiver operating characteristic curve of serum protein carbonyl, uric acid and total bilirubin levels between severe and mild leptospirosis patients. The arrow head shows the cut-off defined (13μmol/mg protein in PC curve and 5.7 mg/ dL in UA curve).

A cut-off value of 13 μmol/ mg protein (the same cut-off considered in SL vs DC comparison) of PC in serum differentiated SL over ML with sensitivity of 80% and specificity of 74% (AUC = 0.78; p<0.001) (Fig 5). Multivariate analysis showed that PC as a marker of severe leptospirosis was independent of age, gender, day of illness or duration of antibiotic therapy. UA and TB levels were significantly elevated in SL compared to ML, presumably reflecting the degree of renal and hepatic involvement seen in patients with severe disease (Fig 2B and 2C). Although both UA and TB levels were significantly high in patients with SL, ROC analysis showed that only UA could be potential in differentiating SL from ML, but not TB. The test had 82% specificity and 60% sensitivity in detecting a SL case over ML case using a cut-off value of 5.7 mg/dL of UA (AUC = 0.77; p<0.001) (Fig 5).

The clinical characteristics, investigations and signs of disease complications observed in patients with SL, ML and DC are presented in Table 2. The criteria of definingdisease complications; myocarditis, acute respiratory distress syndrome, pulmonary haemorrhage, acute liver failure, acute renal injury, shock and multi-organ dysfunction syndrome mentioned in the Table 2 were adopted from Rajapakse etal. 2015 [37]

**Table 2:** Comparison of clinical characteristics and signs of disease complication in patients with severe leptospirosis and mild leptospirosis compared to critical phase dengue.

Clinical investigations and complications	Percentage positivity		
	SL (n = 60)	ML (n = 50)	DC (n = 30)
Fever (%)	100	99.6	100
Headache (%)	100	98.8	100
Myalgia (%)	89.8	78.2	94.3
Conjuctival suffusion (%)	43.8	39.5	0
Myocarditis (%)	8.3	0	0
Acute Respiratory Distress Syndrome (ARDS) (%)	6.6	0	0
Pulmonary haemorrhage (%)	1.6	0	3.33
Jaundice (%)	45.6	9.30	3.33
Acute liver failure (%)	3.3	0	0
Gastro-intestinal bleeding (%)	15.8	2.32	3.33
Acute renal injury (AKI)(%)	100	0	3.33
Haematuria (%)	45.6	14.00	0
Oligurea (%)	65	4.65	0
Shock (%)	20	0	0
Multi-organ dysfunction Syndrome (MODS) (%)	6.6	0	0

Fever, headache and myalgia were common symptoms. All SL patients had acute kidney injury. Other complications seen were myocarditis, ARDS, acute liver failure, shock and multi-organ dysfunction syndrome. No such signs of complications were observed in ML patients. Pulmonary haemorrahage, jaundice, gastro-intestinal bleeding and acute kidney injury were present in some DC patients.

The haematological and biochemical parameters in the three groups of patients are shown in Table 3.

**Table 3:** Haematological and biochemical parameters in SL, ML and DC patients.

Haematological parameters	Mean ± SD		
	SL	ML	DC
Total bilirubin (mg/ dL)	2.24±2.13^a^^,^^b^	0.93±1.01	0.75±0.45
Uric acid (mg/ dL)	7.04±4.04^a^^,^^b^	3.64±2.69	3.98±1.37
Creatinine (μmol/ L)	325.60±178.19^a^^,^^b^	87.66±22.07	33.14±45.84
Sodium (mEq/L)	139.54±4.37	141.40±3.90	136.80±2.58
Potassium (mEq/L)	3.78±0.63	3.58±0.59	4.18±0.24
Alanine transaminase (IU/L)	66.11±57.06	64.33±41.05	229.40±342.30
Alkaline phosphatase (IU/L)	332.80±200.10	251.80±91.40	292±235.20
Aspartate transaminase (IU/L)	98.36±94.63	90.33±73.76	306.4±491.18
Albumin (mg/ dL)	32.82±10.82^b^	35.05±6.77^c^	15.78±16.21
White blood cells (x10^6^)	11.79±5.78^a^^,^^b^	8.28±4.90^c^	5.35±3.73
Neutrophils (%)	74.45±13.87^b^	66.15±17.16^c^	46.59±19.74
Lymphocyte (%)	15.01±9.27^b^	21.43±13.02^c^	44.66±20.05
Haemoglobin (g/dL)	10.53±1.27^a^^,^^b^	12.16±1.76^c^	14.33±1.70
Packed cell volume	36.17±42.80	35.16±6.96	40.96±8.61
Platelets (x10^6^)	114.95±87.15	141.06±86.92^c^	81.17±59.23

^**a**^p<0.05 in mean comparison between SL and ML

^**b**^ p<0.05 between SL and DC and

^**c**^ p<0.05 between ML and DC

Serum albumin levels, white blood cells counts, neutrophil percentages were significantly higher, and haemoglobin levels, and lymphocyte percentages were significantly lower in leptospirosis patients (both SL and ML) compared to DC. SL patients had significantly lower haemoglobin levels and higher white blood cell counts, total bilirubin levels, uric acid levels and creatinine levels compared to ML patients.

### Laboratory parameters and oxidative stress in leptospirosis

Serum PC (r = 0.236, p = 0.024), UA (r = 0.497, p<0.001) and TB(r = 0.235, p = 0.024) levels correlated positively with serum creatinine levels in SL patients. UA levels correlated positively with alanine transaminase (ALP) (r = 0.347, p = 0.006). Positive correlations were seen between WBC (r = 0.269, p = 0.003) and neutrophils (r = 0.243, p = 0.016) with TB. Both UA (r = -0.265, p = 0.008) and TB (r = -0.308, p = 0.002)levels showed a negative correlation with haemoglobin levels.

### Disease complications associated with oxidative stress in leptospirosis

Disease complications and clinical investigations which were significantly correlated with oxidative stress parameters are presented in Table 4.

**Table 4:** Disease complications associated with oxidative stress in patients with leptospirosis.

Disease complication	Oxidative stress parameter		
	PC	UA	TB
AKI	r = 0.356; p<0.001	r = 0.440; p<0.001	r = 0.312; p = 0.001
ALF	NS	NS	r = 0.238; p = 0.013
ARDS	NS	r = 0.211; p = 0.028	r = 0.194; p = 0.043
Pulmonary haemorrhage	NS	NS	r = 0.223; p = 0.02
Shock	NS	NS	r = 0.283; p = 0.003
Jaundice	NS	r = 0.298; p = 0.002	r = 0.351; p<0.001
Haematuria	r = 0.200; p = 0.038	r = 0.293; p = 0.002	r = 0.344; p<0.001
Oliguria	r = 0.316; p = 0.001	r = 0.388; p<0.001	r = 0.281; p = 0.003
Gastro-intestinal bleeding	NS	r = 0.223; p = 0.02	NS

NS–No significant correlation observed

Serum LP and AOC had neither positive nor negative correlation with any of the haematological, biochemical, clinical characteristics or disease complications.

## Discussion

Our findings demonstrate that oxidative damage to proteins and lipids occurs in patients with leptospirosis, evidenced by the presence of elevated PC and LP levels. This is further confirmed by the presence of diminished AOC in patients with leptospirosis, when compared with the healthy controls. An imbalance between pro-oxidants and anti-oxidants resulting from increased amounts of reactive oxygen and nitrogen substrates may be responsible for oxidant induced damage to serum proteins and lipids. We postulate that this increased oxidative stress, reflected by the damage to circulatory proteins and lipids, may play a role in the causation of tissue damage in leptospirosis. Our findings further suggest that significantly higher levels of damage to proteins occur in patients with severe leptospirosis, compared to patients with mild disease. However, LP levels did not appear to be significantly raised in severe leptospirosis compared to mild disease. One possible explanation is that serum contains more proteins and relatively small quantities of lipids, since lipids are mostly present in tissue. PC is formed earlier in response to oxidative damage, and is more stable in serum compared to LP [38]. Thus, we suggest that the high levels of PC indicate that significant oxidative damage to proteins in severe leptospirosis. This oxidative damage to protein may result in secondary impacts beyond damage to cell membrane. Oxidative damage to cellular enzymes can result errors in cell regulation[13].

Measurement of PC levels may have some potential in identifying patients with severe leptospirosis in the early stages, before MAT becomes positive. We studied 13 patients with severe leptospirosis, in whom the MAT done in the acute phase of illness were negative and who were subsequently confirmed to be positive by sero-conversion or four fold rise in titre. PC levels were raised in all of these patients, compared with healthy controls (S2 Table). The limitation of this finding was that we did not compare these patients with patients with similar clinical presentations due to other illnesses; this is a subject for further study.

PC, but not LP, can be used to differentiate severe leptospirosis from mild disease reliably, irrespective of age, gender, day of illness and number of days treated with antibiotics indicating its potentiality as a biomarker of severe disease. Serum UA is also of use in differentiating SL from ML. The positive correlations of PC with creatinine, presence of haematuria and oliguria demonstrate that the increase of PC may be useful as an indicator of renal damage.

Although anti-oxidant capacity was low in leptospirosis compared to healthy controls, there was no difference in anti-oxidant capacity between severe and mild leptospirosis. It is possible that high bilirubin and uric acid levels, which occur as a result of the hepatic and renal dysfunction which are characteristic of severe leptospirosis, act as anti-oxidant buffers. This is supported by the significant positive correlation observed with serum UA levels and with serum PC (r = 0.338; p<0.001) and LP levels (r = 0.235; p = 0.14) among the patients with leptospirosis. Further, the negative correlation of cumulative AOC with PC (r = -0.296; p = 0.002, and LP (r = -0.223; p = 0.02). In other words, increased oxidative stress and loss of renal function occur with the disease severity and level of protein and lipid oxidation is inversely proportionate to cumulative anti-oxidant capacity. Thus, despite there being high levels of oxidative stress in severe leptospirosis, measured anti-oxidant capacity is similar in all degrees of disease severity. Elevated UA levels and TB levels had a positive correlation in CL patients (r = 0.223; p = 0.02).

All patients classified as having severe leptospirosis had acute kidney injury, and all of these patients had elevated PC levels. Thus our study demonstrates, for the first time, that elevated PC levels correlate with disease severity and organ involvement (Table 4). Similarly, high PC levels have been demonstrated in some non-infectious diseases, such as Alzheimer’s disease, respiratory distress syndrome, muscular dystrophy, rheumatoid arthritis, progeria, with increasing age and in some infectious diseases such as malaria, dengue and sepsis compared to healthy controls [39, 40].

In our study, patients in the critical phase of dengue had PC levels similar to healthy controls. This finding is different to the findings of previous studies, where patients with DHF on [38]. This maybe due to differences in the timing of samples, as in the previous study all patients were sampled on day 3 of fever[38], while in our study patients were sampled only during the critical phase of illness. The average day of fever on which sampling was performed in DC patients was 5.3 days (Table 1).

The main limitation in the use of PC, LP and anti-oxidant capacity as markers of oxidative damage to tissues is that they are measured in serum, and thus may not accurately reflect what occurs in the tissues [20]. As mentioned above, PC is the most stable of the measurements, and serum PC, UA and TB levels serve as biomarkers of oxidative stress in severe leptospirosis and high serum levels of them may indicate the loss of cell function due to oxidative degradation in lipids and proteins contribute to the pathogenesis of severe illness. Previous studies are inconsistent about the correlation between serum levels of LP and PC, with some studies showing strong correlation and others showing none [17, 20]. Our study showed a significant correlation between these two parameters in DC patients (r = 0.631; p = 0.002), but no such correlation was seen in leptospirosis patients.

During outbreaks of dengue, leptospirosis cases are misdiagnosed as dengue in endemic regions. Therefore, differentiation of severe leptospirosis patients from critical phase dengue patients is important, especially as both forms are fatal and their management is very different [25, 41]. Clinical features do not reliably differentiate the two conditions. An important and practically useful finding in our study was that PC and LP levels are higher in severe leptospirosis when compared with critical phase dengue. Thus measuring PC and LP levels would be useful in differentiating the two conditions. Gil *et al*. 2004 describes although malondyaldehyde and 4-hydroxyalkenals levels were higher in patients with dengue haemorrhagic fever, total hydroperoxide levels were significantly low [42]. Further, previous studies have shown that serum protein levels are low in patients with severe dengue compared to uncomplicated dengue fever [43], and these findings may explain the low LP and PC levels in critical phase dengue observed in present study.

Identification of biological substances which indicate the disease progression is extremely valuable as it would give evidence for the present condition of the patient compared to the past. Spectrophotometric determination of these parameters would make them more practical to be used in low resource areas where sophisticated instrumentation is not available. Also, the current cost effective techniques are feasible to be carried out in leptospirosis and dengue endemic developing countries to reduce the disease morbidity and mortality. Furthermore, identification of these biomarkers would draw the attention of biomedical scientists to discover more simple but reliable techniques to identify early predictors of severe illness.

Our findings demonstrate that oxidative stress has a role in the pathogenesis of leptospirosis associated with disease complications, and could be mainly due to the significant damage to proteins that occurs in severe disease. Measurement of serum PC levels in patients with leptospirosis may be useful in identifying and predicting those are with severe disease. Serum PC level may also be capable of differentiating severe leptospirosis from critical phase dengue infections. We believe that, these findings would help clinicians to distinguish severe leptospirosis patients, independent of the duration of post infection and the antibiotic treatments to manage them promptly during dengue epidemics.

## Supporting Information

S1 Table Serum parameters of patients and healthy subjects. (DOCX)

S2 Table Protein carbonyl and lipid hydroperoxide levels of severe and mild leptospirosis patients, who had acute MAT titre below the positive cut-off. (DOCX)
