# Supplementary material for: Protein Carbonyl as a Biomarker of Oxidative Stress in Severe Leptospirosis, and Its Usefulness in Differentiating Leptospirosis from Dengue Infections
Source: PLoS One. 2016 Jun 9;11(6):e0156085. doi: 10.1371/journal.pone.0156085 (PMC4900524; doi:10.1371/journal.pone.0156085)
Supplement: S1 Table — (DOCX) [file pone.0156085.s001.docx]

S1 Table. Serum parameters of patients and healthy subjects.

| Serum parameter | CL  (n=110) | SL  (n=60) | ML  (n=50) | DC  (n=30) | HC  (n=30) |
| --- | --- | --- | --- | --- | --- |
| Protein carbonyl  in µmol/ mg protein | 16.1±10.40 | 20.33±10.60 | 11.03±7.48 | 5.61±2.88 | 5.29±1.17 |
| Lipid hydroperoxide  in µM | 15.86±21.03 | 16.56±23.30 | 15.03±18.13 | 0.91 ±2.27 | 0.84±2.83 |
| Total anti-oxidant capacity  in µmol/ mg protein | 38.91±13.76 | 37.37±11.41 | 40.77±16.06 | 37.47±9.31 | 68.2±11.52 |
| Uric acid level  in mg/dL | 5.5±3.87 | 7.04±4.04 | 3.64±2.69 | 3.98±1.32 | 4.22±2.02 |
| Total bilirubin level  in mg/ dL | 1.64±1.83 | 2.24±2.13 | 0.93±1.01 | 0.77±0.46 | 1.23±0.90 |

Data are presented as Mean ± standard deviation. CL- collectively both SL and ML
