# Supplementary material for: Protein Carbonyl as a Biomarker of Oxidative Stress in Severe Leptospirosis, and Its Usefulness in Differentiating Leptospirosis from Dengue Infections
Source: PLoS One. 2016 Jun 9;11(6):e0156085. doi: 10.1371/journal.pone.0156085 (PMC4900524; doi:10.1371/journal.pone.0156085)
Supplement: S2 Table — (DOCX) [file pone.0156085.s002.docx]

S2 Table. Protein carbonyl and lipid hydroperoxide levels of severe and mild leptospirosis patients, who had acute MAT titre below the positive cut-off

|  | Severe leptospirosis | Mild leptospirosis |
| --- | --- | --- |
| No. of patients having acute MAT titre below the cut-off (<400) | 13 | 17 |
| Protein carbonyl level in µmol/ mg protein  (Mean ±SD) | 20.9±10.42 | 9.21±6.16 |
| No. of patients having PC levels above cut-off^a^ | 13 (100%) | 10 (59%) |
| Lipid hydroperoxide level in µM (Mean ±SD) | 14.43±20.27 | 13.87±18.45 |
| No. of patients having LP levels above cut-off^b^ | 7 (54%) | 8 (47%) |

^a^PC cut-off was 7 µmol/ mg protein, ^b^LP cut-off was 0.21µM
